# Prevalence and Associated Lifestyle Factors of Suboptimal Health Status among Chinese Children Using a Multi-Level Model

**DOI:** 10.3390/ijerph17051497

**Published:** 2020-02-26

**Authors:** Tao Xu, Junting Liu, Guangjin Zhu, Shaomei Han

**Affiliations:** 1 Department of Epidemiology and Statistics, Institute of Basic Medical Sciences, Chinese Academy of Medical Sciences and School of Basic Medicine, Peking Union Medical College, Beijing 100005, China; 2 Department of Epidemiology, Capital Institute of Pediatrics, Beijing, 100020, China; winnerljt@163.com; 3 Department of Physiopathology, Institute of Basic Medical Sciences, Chinese Academy of Medical Sciences and School of Basic Medicine, Peking Union Medical College, Beijing 100005, China; basicstat@163.com

**Keywords:** suboptimal health status, prevalence, lifestyle, adolescent

## Abstract

Chinese children are facing health challenges brought by chronic non-communicable diseases, such as physical problems and psychological related health problems. Childhood represents a critical life period when the long-term dietary and lifestyle behaviors are formed. It is necessary to survey the prevalence of suboptimal health status (SHS) among Chinese children and to research the relationship between SHS and lifestyles. This study aimed to examine the prevalence of SHS among Chinese children using a large-scale population survey sample covering school students and nonstudent children, and clarified the relationships between SHS and lifestyle factors using multi-level models controlled for the cluster effect of location and the confounding effect of demographics. Multi-level generalized estimating equation models were used to examine the relationships between SHS and lifestyle factors. Prevalence ratios (PR) and 95% confidence intervals (CI) were used to assess the strength of these relationships. Of the 29,560 children, 14,393 reported one or more SHS symptoms, giving a SHS prevalence of 48.69%. The prevalence of SHS for boys (46.07%) was lower than that for girls (51.05%). After controlling for the cluster effect of living areas and confounding effect of demographic characteristics, lifestyle factors associated with SHS were: less sleep duration, current smokers (PR = 1.085, 95%CI: 1.027–1.147), current drinkers (PR = 1.072, 95%CI: 1.016–1.131), children’ parents suffering from chronic diseases (PR = 1.294, 95%CI: 1.179–1.421), poor sleep quality (PR = 1.470, 95%CI: 1.394–1.550), stress (PR = 1.545, 95%CI: 1.398–1.707), negative life events (PR = 1.237, 95%CI: 1.088–1.406), hypertension (PR = 1.046, 95%CI: 1.009–1.084), unhealthy diet choice (PR = 1.091, 95%CI: 1.051–1.133) and irregular meal time (PR = 1.210, 95%CI: 1.163–1.259). Children who could exercise regularly (PR = 0.897, 95%CI: 0.868–0.927) and those with regular medical checkup (PR = 0.891, 95%CI: 0.854–0.929) were associated with lower prevalence probability of SHS. SHS has become a serious public health challenge for Chinese children. Unhealthy lifestyles were closely associated with SHS. Implementation of preventative strategies are needed to reduce the potential SHS burden associated with these widespread high-risk unhealthy lifestyle behaviors.

## 1. Introduction

With further understanding of health in its broader sense, the definition of health has sensed as not only the absence of disease or infirmity, but also a state of complete physical, mental, and social well-being. Accordingly, suboptimal health status (SHS) are catching more attention among medical professionals as a physical state between health and disease, characterized by declines in vitality, physiological function, and the capacity for adaptation, and including medically undiagnosed or functional somatic syndromes [1,2]. In the past 40 years, China has experienced dramatic changes in social and economic conditions, and Wang et al. considered that these changes would increase the incidences of major chronic diseases [3]. In addition, rapid economic development also meant that more people were facing pressures from work, study and home lives that might develop into SHS. Several previous reports showed that 55%–75% of Chinese adults experienced SHS [4,5,6]. In 2007–2011, we conducted a national survey in six provinces or autonomous regions of China to examine SHS among Chinese adults and children across a broad age range and covering dozens of ethnic minorities. And we found that the prevalence of SHS was 69.46% among Chinese adults and the prevalence for male adults (67.74%) was lower than that for female adults (72.67%) [7]. 

With the transformation of the disease spectrum from predominantly infectious diseases to chronic non-communicable diseases (NCDs), Chinese children are facing health challenges brought by chronic NCDs, such as physical problems (e.g., stunting, obesity [8]) and psychological related health problems. Obesity is a risk factor for an expanding set of chronic diseases, including cardiovascular disease [9,10], diabetes mellitus, chronic kidney disease [9], many cancers [11], and musculoskeletal disorders [12,13]. As research progresses, obesity and related problems are gradually being intervened. However, little attention is paid to mental related health issues, feelings of sadness, anxiety, and somatic symptoms such as headaches in children might be signs of severe emotional problems. Untreated repeated or continuing emotional problems have adverse effects on children’s general, social, and academic development [14,15,16]. Moreover, these difficulties may increase the risk of psychiatric disorders such as anxiety and mood disorder in later life [15,17]. Childhood represent a critical life period when the long-term dietary and lifestyle behaviors are formed. SHS in childhood has detrimental effects on individuals’ health in the long term. Up to date, most of previous SHS studies of Chinese children were based on either only one province or a small sample size and just published on Chinese journals [18,19,20,21]. In addition, previous studies on childhood SHS were always conducted among primary school or middle school students. However, a lot of teenagers might have quit from schools so that school student samples could not reflect general children population. Little was known about the current SHS estimates based on both student sample and non-student children sample. It is necessary to survey the prevalence of SHS among Chinese children and research the relationship between SHS and lifestyle factors.

Furthermore, in multi-stage sampling, survey data have a hierarchical structure in which individuals were nested within higher level sampling units. Individual SHS was determined by not merely individual characteristics (e.g., age, ethnicity, education, and habits at the micro level), but features of the social environments in which individuals live too (e.g., diet habit, living circumstances, culture or school education). Children living in the same areas or studying in the same schools were likely to suffer from the effects of similar living circumstances, developing into a similar health status. Traditional analytical methods such as least square regression and logistic regression assumed that observations were independently but identically distributed. Analyzing multi-level data with traditional analytical methods would result in incorrect inferences in statistical analyses because of violation of these assumptions. Multi-level models provided an appropriate analytical framework to deal with observation dependence in multi-level data [22]. More importantly, multi-level models permit exploration of the nature and extent of relationships at both micro and macro levels, as well as across levels [22]. Specifically, this paper aimed to examine the prevalence of SHS among Chinese children using a large-scale cross-sectional survey sample covering school students and nonstudent children, and clarified the relationships between SHS and lifestyle factors using multi-level generalized estimating equation (GEE) models controlled for the cluster effect of location and the confounding effect of demographics.

## 2. Material and Methods

### 2.1. Sample and Participants

The data was from a large-scale population survey conducted from 2007 to 2011. In brief, this survey was conducted in six provinces or autonomous regions of China to cover a variety of municipal regions and include sufficient minority subjects: Hunan Province, Yunnan Province, Heilongjiang Province, Inner Mongolia Autonomous Region, Sichuan Province, and Ningxia Hui Autonomous Region. In each selected province or autonomous region, a two-stage sampling method was used to recruit eligible subjects. First, two or three cities were selected based on their population and economic conditions using a simple random sampling method. Then, considering that some teenagers might have quit from school, dozens of communities were also selected within each city. All subjects of the selected schools or communities were considered eligible for this study if they were aged 10–17 years, were not suffering from serious chronic diseases, and were not running a high fever in the past 15 days. In each province or autonomous region, about 5000 subjects were chosen, who were of dozens of ethnicities, including Han, Yi, Miao, Mongolian, Tibetan, Korean, Hui, Tujia, and others.

The study was approved by the ethics review board of the Institute of Basic Medical Sciences, Chinese Academy of Medical Sciences (No. 005-2008). Both selected children and their parents signed written informed consent forms. Children carried the informed consent forms to their parents, and then submitted the signed written informed consent forms to medical professionals before the survey. The interview and physical exams were performed by the medical professionals. Both their teachers and adult parents were not present in the interview. This study adhered to strict quality control standards. Trained medical professionals conducted the survey and interviews.

### 2.2. Suboptimal Health Assessment and Definition of Covariates

A Delphi self-rating SHS scale was used to assess the SHS of children [7,23]. The scale included 18 symptom items grouped in six dimensions: physical symptoms, psychological symptoms, vigor, social adaptability, immunity, and going to hospital. 18 symptom items contained fatigue, headache or dizziness, tinnitus, numbness or stiffness in the shoulders or legs, a sense of pharyngeal foreign bodies, upset, loneliness, inattention, anxiety, dreaminess, forgetfulness, decreased vitality, disinterest in surroundings, moodiness, feeling tired at work, incompatibility with coworkers, susceptibility to flu or other diseases and the feeling of suffering from undiagnosed diseases. Children who experienced one or more symptom items for more than one month in the past year were considered as having SHS. Children who did not experienced any symptom items in the past year were considered as being normal health status.

Education was classified into three groups: primary school, junior middle school, and senior middle school. Parents’ diseases indicated whether or not children’ father and/or mother were suffering chronic diseases, such as cardiovascular diseases, cerebrovascular diseases, diabetes, cancer, kidney diseases, etc. According to the fourth report on the diagnosis, evaluation, and treatment of high blood pressure in adolescents [24], hypertension of children was defined as systolic blood pressure and/or diastolic blood pressure levels ≥ 95th percentile for gender, age and height. Percentiles of height were defined according to tables of reports of the physical fitness and health research of Chinese school students [25]. According to the body mass index (BMI) reference norm for Chinese children [26], obesity was defined as BMI ≥ the 95th percentile in the reference. 

Sleep duration and sleep quality were self-reported by children. Sleep duration was their average sleep time per day in the last year, which was classified into >8 h, 6–8 h and <6 h. And sleep quality was dichotomized into good sleep quality and poor sleep quality.

Stress was defined as self-perceived economic, life, or study stress in the past year. Negative life event was defined by whether the child had experienced loss of job, retirement, loss of crops/business failure, burglary, marital separation/divorce, other major intra-family conflict, major personal injury or illness, violence, death of a spouse, death/major illness of another close family member, and other major stressors in the past year. Diet choice was defined as whether the child had unhealthy diet choice preferences, such as food that was highly salty, sweet, spicy, or greasy. Meal time was used to indicate whether the child regularly had breakfast, lunch, and dinner at fixed times or not in the past year. 

### 2.3. Statistical Analysis

All case report forms and database were double-checked to guarantee the authenticity and accuracy of raw data. Statistical analysis was performed with SAS9.4 software (SAS institute Inc., Cary, NC, USA). A two-tailed *p*-value < 0.05 was defined as statistically significant. Continuous data were described using mean and standard deviation. Categorical data were described with number and percentage. Categorical data were compared using chi-square tests. Children living in the same area and studying in the same school were likely to suffer the effects of similar living circumstances and educational culture. Accordingly, multi-level log-binomial GEE models were conducted to examine the relationships between SHS and lifestyle factors to control for the cluster effect of location and the confounding effect of demographic characteristics. Prevalence ratios (PR) and 95% confidence intervals (CI) were used to assess the strength of these relationships.

## 3. Results

In total, 30,071 children signed written informed consent forms and were willing to participate in this survey. Of these, 29,560 children completed all survey scales, giving a completion rate of 98.3%.

The average age of all subjects was 13.8 ± 2.3 years, and 52.61% of subjects were girls. The majority of all subjects (73.28%) were of Han nationality. The percentages of current smokers and drinkers were 3.95% and 3.22%, respectively. The percentages of obese and hypertensive children were 4.50% and 4.13%, respectively. In addition, 567 children reported feeling high levels of study, home, or economic stress, and 1513 children had recently experienced negative life events. Just under half (45.00%) of the children took part in medical checkup regularly and 63.80% of the children exercised regularly. Just half (50.19%) of the children slept for >8 h per days and only one-third of children (35.37%) reported good sleep quality. The majority of children (60.41%) had unhealthy dietary choices and 11.10% of children did not have meals at fixed hours regularly. Further, about one-eighth of all children reported that their father and/or mother were suffering from chronic diseases.

Of the 29,560 children, 14,393 reported one or more SHS symptoms, giving a SHS prevalence of 48.69%. The most common SHS symptoms were inattention (26.38%), forgetfulness (19.24%), and upset (18.13%). Table 1 shows the prevalence of the 18 SHS symptoms for the whole sample, and by gender and age groups.

The average age of children with SHS was 14.6 ± 2.1 years and that of children without SHS was 13.1 ± 2.3 years. The prevalence of SHS was 22.59% for subjects aged 10–11 years, 43.39% for those aged 12–13 years, 59.35% for those aged 14–15 years and 67.32% for those aged 16–17 years. The prevalence of SHS for boys (46.07%) was lower than that for girls (51.05%) (*p* < 0.0001). The prevalence of SHS was significantly different between ethnicities: children from Tujia had the lowest prevalence of SHS (38.65%) and those from Yi had the highest prevalence (69.83%). The prevalence of SHS increased as educational level increased (*p* < 0.0001). 

Children whose parents were suffering from chronic diseases had a higher prevalence of SHS than their counterparts (72.13% vs. 45.21%). Compared with children with normal blood pressure, hypertensive children had a higher prevalence of SHS (54.95% vs. 48.42%). Current smokers (65.12% vs. 48.02%) or alcohol consumers (69.40% vs. 48.00%) had a higher prevalence of SHS than their counterparts. We observed higher prevalence rates of SHS among children with poor sleep quality compared with their counterparts (54.27% vs. 38.50%). The prevalence rate of SHS was 38.60%, 56.46% and 79.92% for subjects with >8 h of sleep, 6–8 h of sleep and <6 h of sleep per day respectively. Children who reported experiencing stress had a higher prevalence of SHS than their counterparts (88.89% vs. 47.90%), as did those who had experienced negative life events (78.39% vs. 47.09%). Children who had unhealthy diet choices had a higher prevalence of SHS than their counterparts (52.05% vs. 43.57%). Children who exercised regularly had a lower prevalence of SHS than their counterparts (44.82% vs. 55.50%), as did those who had regular medical checkup (38.75% vs. 56.82%), and those who had regular meals at fixed hours (47.06% vs. 61.75%). The prevalence of SHS by different demographic characteristics and lifestyle factors are detailed in Table 2. 

Table 3 presents the results of the univariate and multivariate multi-level GEE models factors associated with SHS. After controlling for the cluster effect of location and the confounding effect of demographics, we found that age, gender, education, smoking, alcohol drinking, ethnicity, parents’ suffering from chronic diseases, sleep duration, sleep quality, stress, negative life events, exercise, hypertension, medical checkup, exercise, diet choice, and meal times were associated with SHS. Compared to children aged 10–11 years, children aged 14–15 years (PR = 1.349, 95%CI: 1.202–1.513) and 16–17 years (PR = 1.372, 95%CI: 1.213–1.552) were associated with higher prevalence probability of SHS. Compared to boys, girls (PR = 1.047, 95%CI: 1.019–1.075) were associated with a higher prevalence probability of SHS. Compared to children in primary school, children in junior middle school (PR = 1.221, 95%CI: 1.042–1.431) and senior middle school (PR = 1.208, 95%CI: 1.035–1.408) were associated with higher prevalence probability of SHS. Compared to Hans, Koreans (PR = 1.602, 95%CI: 1.305–1.966), Huis (PR = 1.114, 95%CI: 1.069–1.161), and Yis (PR = 1.059, 95%CI: 1.005–1.116) had a higher prevalence probability of SHS, however Tibetans (PR = 0.747, 95%CI: 0.524–1.064) had a lower prevalence probability.

Compared to children sleeping more than 8 h per day, children sleeping 6–8 h per day (PR = 1.039, 95%CI: 1.000–1.079) or those sleeping less than 6 h per day (PR = 1.228, 95%CI: 1.165–1.296) had higher prevalence probability of SHS. Current smokers (PR = 1.085, 95%CI: 1.027–1.147), current drinkers (PR = 1.072, 95%CI: 1.016–1.131), children whose parents were suffering from chronic diseases (PR = 1.294, 95%CI: 1.179–1.421), children with poor sleep quality (PR = 1.470, 95%CI: 1.394–1.550), children who felt about some stress (PR = 1.545, 95%CI: 1.398–1.707) or negative life events (PR = 1.237, 95%CI: 1.088–1.406), hypertensive children (PR = 1.046, 95%CI: 1.009–1.084), children who had unhealthy diet choice (PR = 1.091, 95%CI: 1.051–1.133) and children who could not have breakfast, lunch and dinner at fixed hours regularly (PR = 1.210, 95%CI: 1.163–1.259) had higher prevalence probability of SHS. Children who could exercise regularly (PR = 0.897, 95%CI: 0.868–0.927) and those who could take part in medical checkup regularly (PR = 0.891, 95%CI: 0.854–0.929) were associated with lower prevalence probability of SHS. 

We excluded those who suffered from serious chronic diseases and retained those with mild hypertension (1211 children) in our study. A sensitivity analysis where those with hypertension were excluded was conducted and the findings of multivariate analysis are shown in Table 3.

## 4. Discussion

Previous studies ever reported the prevalence of SHS among college students aged above 18 years old [5,27,28,29,30]. But little was reported about prevalence of SHS among children aged 10–17 years old. In this study we found that about half of Chinese children aged from 10–17 years old had SHS. This was the first study to examine the prevalence of SHS among Chinese children by a population sample covering school students and nonstudent children. Moreover, this is the first time to examine the relationships of childhood SHS and lifestyle factors using multi-level GEE models to control for the cluster effect of the same living area and similar living circumstances.

Lifestyle was one of the most important factors affecting diseases and unhealthy lifestyles were closely related with many chronic diseases [31,32,33,34]. Chen et al. considered that SHS was highly attributable to unhealthy lifestyles, and the mitigation of modifiable lifestyle risk factors may lead to SHS regression for college students [28]. Several studies also found that many lifestyle factors were closely associated with SHS among adults [5,29,30,35]. In our previous study, unhealthy lifestyles were found closely associated with SHS among adults aged above 18 years old, including smoking, drinking alcohol, short sleep duration, poor sleep quality, lack of regular exercise, stress, negative life events, unhealthy diet choices, and irregular meal times [7]. In this study, we found that these lifestyle factors were also closely associated with SHS among children aged 10–17 years old. 

Tobacco smoking and drinking alcohol have been confirmed as associated with many chronic diseases [36,37,38,39], and to adversely affect the health of children [40,41]. Therefore, more effective measurements should be conducted to make children more aware of the harm of smoking and alcohol drinking on health. Sleep deprivation and sleep disruptions were reported to be able to cause severe cognitive and emotional problems [42,43]. >8 h of sleep per day was referred to as adequate sleep for children according to two previous reports [44,45]. However, just half of the children could sleep for >8 h per day and only one-third of children reported good sleep quality in this study. Sleep duration has been considered gradually declining over the past decades [46]. Adequate sleep was very important to optimal daily function and behavior in children. To promote health and to prevent and manage sleep problems, it was necessary to understand the factors that affected children sleep. Children sleep were influenced by not only the children factors, but also the parent factors and the environmental factors. A growing proportion of children were curtailing their sleep duration in response to increasing demands and lifestyle changes, such as prolonged studying hours, and introduction of new electronic technologies. Short sleep duration and poor sleep quality were found consistently associated with the Internet use, mobile phone use and number of devices in the bedroom [47,48,49,50]. Public health efforts that encouraged children to have sufficient sleep may be important in preventing SHS by decreasing studying burden and dependency on electronic devices.

If their parents were suffering from chronic diseases, children were more anxious and in worse economic conditions which could in turn make them more susceptible to SHS. Economic, life, or study stress and negative life events were important factors for depression and anxiety [51,52], and could further lead to the incidence of SHS. Moreover, early negative life event and stress were reported associated with physiological and psychological diseases in later life [53,54,55]. Low levels of physical activity were known as risk factors for obesity, depression, anxiety, self-esteem and cognitive functioning in children [56,57,58]. Guaranteeing children to get enough physical activity and regular exercise would help improve health of children. Furthermore, taking part in medical checkup regularly could discover and treat diseases in time, which were contributable to keep healthy for children.

A systematic review reported ever that there was a relationship between unhealthy diet, consumption of low-quality diet and depression or poor mental health [59]. In our study, unhealthy diet choices, and irregular meal times were also found as associated with a higher prevalence risk of SHS. Make sure that children had healthy diet choices and had breakfast, lunch and dinner at fixed times regularly, which could help them to maintain healthy living habits and then benefit their physical and psychological health. Hypertension of children should also be paid more attention. Since some of the SHS symptoms (e.g., headache and dizziness) may be caused by hypertension, a sensitivity analysis was conducted in which those with hypertension were excluded. The sensitivity analysis showed that there was no significant difference of the study findings between among all children and among children without hypertension. This indicated that hypertension has no significant confounding effect on the study finding of other covariates. 

This study had several limitations that should be mentioned. First, because of the cross-sectional design, it was not possible to confirm causal relationships between SHS and lifestyle factors. There was a possibility that both some lifestyle factors and SHS resulted from some other predictors simultaneously. Further, even children with SHS may not have enough energy to engage in physical activity, thus leading to inadequate physical activity. Therefore, we could conclude that unhealthy lifestyles were closely associated with SHS, but not confirm the independent prediction effect of unhealthy lifestyle on SHS. Second, considering that low-aged children had difficulty understanding the question items of the SHS scale, we didn’t enroll children aged less than ten years old. Third, since the basic data of this study were obtained 10 years ago and the health problems and factors of Chinese children are changing, the study findings reflected the situation at that time, and may not reflect current situation. Finally, some data were self-reported, such as smoking, alcohol drinking, sleep, diet in this study, and such self-reported knowledge reduced the precision of lifestyle measurement.

## 5. Conclusions

Despite these limitations, our findings showed that SHS has become a serious public health challenge for Chinese children. Unhealthy lifestyles were closely associated with SHS, with key factors being smoking, drinking alcohol, short sleep duration, poor sleep quality, lack of regular checkup, inadequate physical activity, stress, negative life events, parents suffering from chronic diseases, unhealthy diet choices, and irregular meal times. Implementation of preventative strategies are needed to reduce the potential SHS burden associated with these widespread high-risk unhealthy lifestyle behaviors.

## Figures and Tables

**Table 1:** Prevalence (%) of 18 suboptimal health symptom items by gender and age groups.

Symptom Item	Total (n = 29,560)	Gender		Age Groups			
		Boy (n = 14,009)	Girl (n = 15,551)	10–11 y (n = 7007)	12–13 y (n = 7417)	14–15 y (n = 7499)	16–17 y (n = 7637)
fatigue	5776 (19.54%)	1830 (13.06%)	2828 (18.19%)	348 (4.97%)	1142 (15.40%)	1892 (25.23%)	2394 (31.35%)
headache or dizziness	4658 (15.76%)	693 (4.95%)	793 (5.10%)	525 (7.49%)	1082 (14.59%)	1464 (19.52%)	1587 (20.78%)
tinnitus	1486 (5.03%)	693 (4.95%)	793 (5.10%)	132 (1.88%)	302 (4.07%)	490 (6.53%)	562 (7.36%)
numbness or stiffness in the shoulders or legs	1697 (5.74%)	748 (5.34%)	949 (6.10%)	135 (1.93%)	371 (5.00%)	563 (7.51%)	628 (8.22%)
a sense of pharyngeal foreign bodies	2884 (9.76%)	1426 (10.18%)	1458 (9.38%)	235 (3.35%)	595 (8.02%)	947 (12.63%)	1107 (14.50%)
upset	5359 (18.13%)	2270 (16.20%)	3089 (19.86%)	349 (4.98%)	1034 (13.94%)	1753 (23.38%)	2223 (29.11%)
loneliness	3292 (11.14%)	1383 (9.87%)	1909 (12.28%)	284 (4.05%)	614 (8.28%)	1064 (14.19%)	1330 (17.42%)
inattention	7798 (26.38%)	3589 (25.62%)	4209 (27.07%)	680 (9.70%)	1621 (21.86%)	2524 (33.66%)	2973 (38.93%)
anxiety	3641 (12.32%)	1570 (11.21%)	2071 (13.32%)	198 (2.83%)	628 (8.47%)	1180 (15.74%)	1635 (21.41%)
dreaminess	4956 (16.77%)	2004 (14.31%)	2952 (18.98%)	357 (5.09%)	940 (12.67%)	1553 (20.71%)	2106 (27.58%)
forgetfulness	5688 (19.24%)	2470 (17.63%)	3218 (20.69%)	426 (6.08%)	1011 (13.63%)	1845 (24.60%)	2406 (31.50%)
decreased vitality	2754 (9.32%)	1227 (8.76%)	1527 (9.82%)	177 (2.53%)	480 (6.47%)	897 (11.96%)	1200 (15.71%)
disinterest in surroundings	3342 (11.31%)	1508 (10.76%)	1834 (11.79%)	263 (3.75%)	625 (8.43%)	1063 (14.18%)	1391 (18.21%)
moodiness	4209 (14.24%)	1754 (12.52%)	2455 (15.79%)	278 (3.97%)	747 (10.07%)	1356 (18.08%)	1828 (23.94%)
feeling tired at work	2454 (8.30%)	1169 (8.34%)	1285 (8.26%)	118 (1.68%)	427 (5.76%)	872 (11.63%)	1037 (13.58%)
incompatibility with coworkers	1009 (3.41%)	462 (3.30%)	547 (3.52%)	97 (1.38%)	202 (2.72%)	327 (4.36%)	383 (5.02%)
susceptibility to flu or other diseases	3832 (12.96%)	1528 (10.91%)	2304 (14.82%)	404 (5.77%)	825 (11.12%)	1245 (16.60%)	1358 (17.78%)
the feeling of suffering from undiagnosed diseases	2019 (6.83%)	881 (6.29%)	1138 (7.32%)	114 (1.63%)	362 (4.88%)	696 (9.28%)	847 (11.09%)

**Table 2:** Prevalence (%) of suboptimal health by adolescent’ demographic characteristics.

	Total	Suboptimal Health Status		Chi-Square Test	
		No	Yes	Chi-Square	*p* *
All subjects	29,560	15,167 (51.31%)	14,393 (48.69%)		
Age (years)				3395.8779	<0.0001
10–11	7007	5424 (77.41%)	1583 (22.59%)		
12–13	7417	4199 (56.61%)	3218 (43.39%)		
14–15	7499	3048 (40.65%)	4451 (59.35%)		
16–17	7637	2496 (32.68%)	5141 (67.32%)		
Gender				73.1899	<0.0001
Boy	14,009	7555 (53.93%)	6454 (46.07%)		
Girl	15,551	7612 (48.95%)	7939 (51.05%)		
Education level				2766.8542	<0.0001
Primary school	8804	6550 (74.40%)	2254 (25.60%)		
Junior middle school	11,764	5225 (44.42%)	6539 (55.58%)		
Senior middle school	8992	3392 (37.72%)	5600 (62.28%)		
Smoker				131.3347	<0.0001
No	28,393	14,760 (51.98%)	13,633 (48.02%)		
Yes	1167	407 (34.88%)	760 (65.12%)		
Alcohol drinker				168.6915	<0.0001
No	28,609	14,876 (52.00%)	13,733 (48.00%)		
Yes	951	291 (30.60%)	660 (69.40%)		
Ethnicity				542.0118	<0.0001
Han	21,663	11,376 (52.51%)	10,287 (47.49%)		
Tibetan	880	446 (50.68%)	434 (49.32%)		
Korean	1149	758 (65.97%)	391 (34.03%)		
Hui	1778	687 (38.64%)	1091 (61.36%)		
Mongolia	1203	619 (51.45%)	584 (48.55%)		
Miao	490	322 (65.71%)	168 (34.29%)		
Tujia	414	254 (61.35%)	160 (38.65%)		
Yi	1445	436 (30.17%)	1009 (69.83%)		
Others	538	269 (50.00%)	269 (50.00%)		
Parents’ diseases				966.2329	<0.0001
No	25,735	14,101 (54.79%)	11,634 (45.21%)		
Yes	3825	1066 (27.87%)	2759 (72.13%)		
Obesity				61.9002	<0.0001
No	28,231	14,345 (50.81%)	13,886 (49.19%)		
Yes	1329	822 (61.85%)	507 (38.15%)		
Sleep duration				1385.0135	<0.0001
>8 h	14,835	9109 (61.40%)	5726 (38.60%)		
6–8 h	12,280	5347 (43.54%)	6933 (56.46%)		
<6 h	2445	711 (2908%)	1734 (70.92%)		
Sleep quality				671.9875	<0.0001
Good	10,456	6430 (61.50%)	4026 (38.50%)		
Poor	19,104	8737 (45.73%)	10,367 (54.27%)		
Stress				373.9065	<0.0001
No	28,993	15,104 (52.10%)	13,889 (47.90%)		
Yes	567	63 (11.11%)	504 (88.89%)		
Negative life event				562.8928	<0.0001
No	28,047	14,840 (52.91%)	13,207 (47.09%)		
Yes	1513	327 (21.61%)	186 (78.39%)		
Regular check-up				955.9663	<0.0001
No	16,258	7020 (43.18%)	9238 (56.82%)		
Yes	13,302	8147 (61.25%)	5155 (38.75%)		
Hypertension				20.0041	<0.0001
No	28,339	14,617 (51.58%)	13,722 (48.42%)		
Yes	1221	550 (45.05%)	671 (54.95%)		
Regular exercise				311.6655	<0.0001
No	10,702	4762 (44.50%)	5940 (55.50%)		
Yes	18,858	10,405 (55.18%)	8453 (44.82%)		
Diet choice				203.3397	<0.0001
Routine	11,703	6604 (56.43%)	5099 (43.57%)		
Unhealthy	17,857	8563 (47.95%)	9294 (52.05%)		
Meal time				251.9170	<0.0001
Regular	26,729	13,912 (52.94%)	12,367 (47.06%)		
Irregular	3281	1255 (38.25%)	2026 (61.75%)		

* *p* value for Chi-square test.

**Table 3:** Risk factors associated with suboptimal health based on a multi-level GEE model.

Characteristics	All Children				Children without Hypertension	
	Univariate		Multivariate		Multivariate	
	PR	95%CI	PR	95%CI	PR	95%CI
Age (years)						
10–11	1.000	-	1.000	-	1.000	-
12–13	2.626	2.443–2.824	1.057	0.945–1.181	1.323	1.050–1.669
14–15	5.004	4.655–5.381	1.349	1.202–1.503	1.630	1.338–1.986
16–17	7.057	6.558–7.598	1.372	1.213–1.552	1.693	1.393–2.059
Gender						
Boy	1.000	-	1.000	-	1.000	-
Girl	1.221	1.166–1.278	1.047	1.019–1.075	1.067	1.000–1.143
Education level						
Primary school	1.000	-	1.000	-	1.000	-
Junior middle school	3.637	3.425–3.863	1.221	1.042–1.431	1.985	1.155–3.412
Senior middle school	4.798	4.500–5.116	1.208	1.035–1.408	1.851	1.125–3.045
Smoker						
No	1.000	-	1.000	-	1.000	-
Yes	2.022	1.790–2.287	1.085	1.027–1.147	1.223	1.088–1.373
Alcohol drinker						
No	1.000	-	1.000	-	1.000	-
Yes	2.456	2.139–2.829	1.072	1.016–1.131	1.101	1.009–1.201
Ethnicity						
Han	1.000	-	1.000	-	1.000	-
Tibetan	1.076	0.940–1.231	0.747	0.524–1.064	0.751	0.515–1.906
Korean	0.570	0.503–0.646	1.602	1.305–1.966	1.586	1.197–2.101
Hui	1.756	1.591–1.940	1.114	1.069–1.161	1.296	1.177–1.428
Mongolia	1.043	0.929–1.172	1.006	0.956–1.060	0.895	0.773–1.036
Miao	0.577	0.477–0.695	0.986	0.831–1.170	0.839	0.658–1.069
Tujia	0.697	0.570–0.849	1.074	0.900–1.280	1.082	0.846–1.383
Yi	2.558	2.282–2.875	1.059	1.005–1.116	0.958	0.896–1.025
Others	1.106	0.932–1.312	1.034	0.984–1.087	1.046	0.935–1.169
Parents’ diseases						
No	1.000	-	1.000	-	1.000	-
Yes	3.137	2.912–3.382	1.294	1.179–1.421	1.517	1.363–1.689
Obesity						
No	1.000	-	1.000		1.000	-
Yes	0.637	0.569–0.713	1.001	0.934–1.073	1.015	0.927–1.111
Sleep duration						
>8 h	1.000	-	1.000	-	1.000	-
6–8 h	2.063	1.965–2.166	1.039	1.000–1.079	1.068	0.944–1.208
<6 h	3.877	3.536–4.261	1.228	1.165–1.296	1.320	1.183–1.473
Sleep quality						
Good	1.000	-	1.000	-	1.000	-
Poor	1.895	1.805–1.990	1.470	1.394–1.550	1.476	1.359–1.603
Stress						
No	1.000	-	1.000	-	1.000	-
Yes	8.700	6.745–11.422	1.545	1.398–1.707	1.577	1.330–1.870
Negative life event						
No	1.000	-	1.000	-	1.000	-
Yes	4.075	3.603–4.623	1.237	1.088–1.406	1.218	1.036–1.536
Regular check-up						
No	1.000	-	1.000	-	1.000	-
Yes	0.481	0.459–0.504	0.891	0.854–0.929	0.835	0.773–0.901
Hypertension						
No	1.000	-	1.000	-	-	-
Yes	1.299	1.158–1.458	1.046	1.009–1.084	-	-
Regular exercise						
No	1.000	-	1.000	-	1.000	-
Yes	0.651	0.621–0.683	0.897	0.868–0.927	0.871	0.816–0.930
Diet choice						
Routine	1.000	-	1.000	-	1.000	-
Unhealthy	1.406	1.341–1.473	1.091	1.051–1.133	1.207	1.101–1.322
Meal time						
Regular	1.000	-	1.000	-	1.000	-
Irregular	1.816	1.686–1.957	1.210	1.163–1.259	1.322	1.258–1.390

PR: Prevalence ratio; CI: confidence interval; GEE: generalized estimating equation.

## References

[1] China Association of Chinese Medicine (2006). The Traditional Chinese Medicine Clinical Guidelines of Suboptimal Health Status.

[2] Wang W., Yan Y. (2012). Suboptimal health: A new health dimension for translational medicine. Clin. Transl. Med..

[3] Wang L., Kong L., Wu F., Bai Y., Burton R. (2005). Preventing chronic diseases in China. Lancet.

[4] Wang D., Zhou H. (2007). Healthy, disease and sub-healthy. Med. Soc..

[5] Ma C., Xu W., Zhou L., Ma S., Wang Y. (2018). Association between lifestyle factors and suboptimal health status among Chinese college freshmen: A cross-sectional study. BMC Public Health.

[6] Xie Y., Liu B., Piao H. (2006). Exploration on the common characters of sub-healthy people based on clinical epidemiology. J. Chin. Integr. Med..

[7] Xu T., Zhu G., Han S. (2020). Prevalence of suboptimal health status and the relationships between suboptimal health status and lifestyle factors among Chinese adults using a multi-level generalized estimating equation model. Int. J. Environ. Res. Public Health.

[8] GBD 2015 Obesity Collaborators (2017). Health effects of overweight and obesity in 195 countries over 25 years. N. Engl. J. Med..

[9] Singh G.M., Danaei G., Farzadfar F., Stevens G.A., Woodward M., Wormser D., Kaptoge S., Whitlock G., Qiao Q., Lewington S. (2013). The Age-specific quantitative effects of metabolic risk factors on Cardiovascular diseases and diabetes: A pooled analysis. PLoS ONE.

[10] Wormser D., Kaptoge S., Di Angelantonio E., Wood A.M., Pennells L., Thompson A., Sarwar N., Kizer J.R., Lawlor D.A., Emerging Risk Factors Collaboration (2011). Separate and combined associations of body-mass index and abdominal adiposity with cardiovascular disease: Collaborative analysis of 58 prospective studies. Lancet.

[11] Lauby-Secretan B., Scoccianti C., Loomis D., Grosse Y., Bianchini F., Straif K. (2016). Body fatness and cancer-viewpoint of the IARC Working Group. N. Engl. J. Med..

[12] Jiang L., Rong J., Wang Y., Hu F., Bao C., Li X., Zhao Y. (2011). The relationship between body mass index and hip osteoarthritis: A systematic review and meta-analysis. Jt. Bone Spine.

[13] Jiang L., Tian W., Wang Y., Rong J., Bao C., Liu Y., Zhao Y., Wang C. (2012). Body mass index and susceptibility to knee osteoarthritis: A systematic review and meta-analysis. Jt. Bone Spine.

[14] Davis T.E., Ollendick T.H., Nebel-Schwalm M. (2008). Intellectual ability and achievement in anxiety-disordered children: A clarification and extension of the literature. J. Psychopathol. Behav..

[15] Woodward L.J., Fergusson D.M. (2011). Life course outcomes of young people with anxiety disorders in adolescence. J. Am. Acad. Child Adolesc. Psychiatry.

[16] Nolen-Hoeksema S., Girgus J.S., Seligman M.E. (1992). Predictors and consequences of childhood depressive symptoms: A 5-year longitudinal study. J. Abnorm. Psychol..

[17] Roza S.J., Hofstra M.B., Van der Ende J. (2003). Stable prediction of mood and anxiety disorders based on behavioral and emotional problems in childhood: A 14-year follow-up during childhood, adolescence, and young adulthood. Am. J. Psychiatry..

[18] Liang L., Li L., Zhou Y., Jia R., Wang Y. (2016). Psychological Sub-health Status and the Effect of the Childhood Abuse on It among Middle School Students in Zhengzhou. China J. Health Psychol..

[19] Zhao Y., Yu M., Zhao Z., Ma S., Wan Y. (2019). Study on the association between screen time and physical as well as psychological sub-health in middle school students. Chin. J. Child Health Care.

[20] Wang Y., Wang H., Li J., Liu Q., Cao X. (2017). Sub-health status of middle school students of different modes of care-taking in three-Gorge area of chongqing. Chin. Gen. Pract..

[21] Ji C., Luo W., Hu Z. (2013). Statues of sub-health and its relationship with family and school atmosphere among 460 middle school students in Taiyuan. Chin. J. Child Health Care.

[22] Wang J., Xie H., Fisher J.H. (2010). Multilevel Models Applications Using SAS.

[23] Chen Q., Wang S., Jing C., Dong X., Chi G., Zhu L. (2003). Evaluation on diagnostic criterion of suboptimal health with Delphi method. Chin. J. Public Health.

[24] National High Blood Pressure Education Program Working Group on High Blood Pressure in Adolescents (2004). The fourth report on the diagnosis, evaluation, and treatment of high blood pressure in adolescents. Pediatrics.

[25] Group of Physical Fitness and Health Research of Chinese School Students (2012). Reports of the Physical Fitness and Health Research of Chinese School Students.

[26] Group of China Obesity Task Force (2004). Body mass index reference norm for screening overweight and obesity in Chinese adolescents. Chin. J. Epidemiol..

[27] Hou H., Feng X., Li Y., Meng Z., Guo D., Wang F., Guo Z., Zheng Y., Peng Z., Zhang W. (2018). Suboptimal health status and psychological symptoms among Chinese college students: A perspective of predictive, preventive and personalised health. EPMA J..

[28] Chen J., Xiang H., Jiang P., Yu L., Jing Y., Li F., Wu S., Fu X., Liu Y., Kwan H. (2017). The Role of Healthy Lifestyle in the Implementation of Regressing Suboptimal Health Status among College Students in China: A Nested Case-Control Study. Int. J. Environ. Res. Public Health..

[29] Bi J., Huang Y., Xiao Y., Cheng J., Li F., Wang T., Chen J., Wu L., Liu Y., Luo R. (2014). Association of lifestyle factors and suboptimal health status: A cross-sectional study of Chinese students. BMJ Open.

[30] Chen J., Yu K., Sun X., Chen Z., Kuang L., Ji Y., Zhao X., Luo R. (2016). Effect of health-promoting lifestyle on outcomes of suboptimal health status. J. South. Med. Univ..

[31] Folsom A.R., Yatsuya H., Nettleton J.A., Lutsey P.L., Cushman M., Rosamond W.D., ARIC Study Investigators (2011). Community prevalence of ideal cardiovascular health, by the American Heart Association definition, and relationship with cardiovascular disease incidence. J. Am. Coll. Cardiol..

[32] Sikorski C., Luppa M., Weyerer S., König H.H., Maier W., Schön G., Petersen J.J., Gensichen J., Fuchs A., Bickel H. (2014). Obesity and associated lifestyle in a large sample of Multi- Morbid German primary care attendees. PLoS ONE.

[33] Okami Y., Kato T., Nin G., Harada K., Aoi W., Wada S., Higashi A., Okuyama Y., Takakuwa S., Ichikawa H. (2011). Lifestyle and psychological factors related to irritable bowel syndrome in nursing and medical school students. J. Gastroenterol..

[34] Lin C.C., Li C.I., Liu C.S., Lin W.Y., Fuh M.M.T., Yang S.Y., Lee C.C., Li T.C. (2012). Impact of lifestyle-related factors on all- cause and cause- specific mortality in patients with type 2 diabetes the taichung diabetes study. Diabetes Care.

[35] Wu S., Xuan Z., Li F., Xiao W., Fu X., Jiang P., Chen J., Xiang L., Liu Y., Nie X. (2016). Work-recreation balance, health-promoting lifestyles and SHS in southern China: A cross-sectional study. Int. J. Environ. Res Public Health.

[36] Cohen S.M. (2016). Alcoholic Liver Disease. Clin. Liver Dis..

[37] Sasco A.J., Secretan M.B., Straif K. (2004). Tobacco smoking and cancer: A brief review of recent epidemiological evidence. Lung Cancer.

[38] Erol A., Karpyak V.M. (2015). Sex and gender-related differences in alcohol use and its consequences: Contemporary knowledge and future research considerations. Drug Alcohol Depend..

[39] Akter S., Goto A., Mizoue T. (2017). Smoking and the risk of type 2 diabetes in Japan: A systematic review and meta-analysis. J. Epidemiol..

[40] Makadia L.D., Roper P.J., Andrews J.O., Tingen M.S. (2017). Tobacco Use and Smoke Exposure in Children: New Trends, Harm, and Strategies to Improve Health Outcomes. Curr. Allergy Asthma Rep..

[41] Vrkić B.I., Vrca A., Saraga M. (2018). Changing Pattern of Acute Alcohol Intoxications in Children. Med. Sci. Monit..

[42] Brown L.K. (2012). Can sleep deprivation studies explain why human adolescents sleep?. Curr. Opin. Pulm. Med..

[43] Killgore W.D.S. (2010). Effects of sleep deprivation on cognition. Prog. Brain Res..

[44] Song C., Gong W., Ding C., Zhang Y. (2017). Sleep duration among Chinese children and adolescents aged 6–17 years old. Chin. J. Sch. Health.

[45] Huang G., Hou D., Gao A., Zhu Z., Yu Z., Lin N., Chang S., Mi J. (2018). The analysis of the association of sleep with high blood pressure among children and adolescents aged 6–16 years in Beijing. Chin. J. Prev. Med..

[46] Bin Y.S., Marshall N.S., Glozier N. (2012). Secular trends in adult sleep duration: A systematic review. Sleep Med. Rev..

[47] Bruni O., Sette S., Fontanesi L., Baiocco R., Laghi F., Baumgartner E. (2015). Technology use and sleep quality in preadolescence and adolescence. J. Clin. Sleep Med..

[48] Thomée S. (2018). Mobile Phone Use and Mental Health. A review of the research that takes a psychological perspective on exposure. Int. J. Environ. Res Public Health..

[49] Akçay D., Akçay B.D. (2018). The influence of media on the sleep quality in adolescents. Turk. J. Pediatr..

[50] LeBourgeois M.K., Hale L., Chang A.M., Akacem L.D., Montgomery-Downs H.E., Buxton O.M. (2017). Digital Media and Sleep in Childhood and Adolescence. Pediatrics.

[51] Stikkelbroek Y., Bodden D.H., Kleinjan M., Reijnders M., Van A.L. (2016). Adolescent depression and negative life Events, the mediating role of cognitive emotion regulation. PLoS ONE.

[52] Zou P., Sun L., Yang W., Zeng Y., Chen Q., Yang H., Zhou N., Zhang G., Liu J., Li Y. (2018). Associations between negative life events and anxiety, depressive, and stress symptoms: A cross-sectional study among Chinese male senior college students. Psychiatry Res..

[53] Wang X., Dong Z., Zhan T., Jiang Y., Chen Y., Xu S. (2019). Effect of early adverse life events on adult irritable bowel syndrome. Chin. J. Gastroenterol..

[54] McGinnis D. (2018). Resilience, life events, and well-Being during midlife: Examining resilience subgroups. J. Adult Dev..

[55] Guo C., Zhao Y., Yang F. (2019). Long-term effects of early-life disaster exposure on mental health during the whole life cycle. Chin. J. Dis. Control Prev..

[56] Mokabane N.N., Mashao M.M., Van S.M., Potgieter M., Potgieter A. (2014). Low levels of physical activity in female adolescents cause overweight and obesity: Are our schools failing our children?. S. Afr. Med. J..

[57] Korczak D.J., Madigan S., Colasanto M. (2017). Children’s physical activity and depression: A meta-analysis. Pediatrics.

[58] Biddle S.J., Asare M. (2011). Physical activity and mental health in children and adolescents: A review of reviews. Br. J. Sports Med..

[59] Khalid S., Williams C.M., Reynolds S.A. (2016). Is there an association between diet and depression in children and adolescents? A systematic review. Br. J. Nutr..

